# Nature of dispensing errors in selected hospitals providing free healthcare: a multi-center study in Sri Lanka

**DOI:** 10.1186/s12913-020-05968-y

**Published:** 2020-12-14

**Authors:** R. A. N. Dilsha, H. M. I. P. Kularathne, M. T. M. Mujammil, S. M. M. Irshad, N. R. Samaranayake

**Affiliations:** 1 Department of Pharmacy, Faculty of Health Sciences, The Open University of Sri Lanka, Nugegoda, Sri Lanka; 2 Department of Pharmacy and Pharmaceutical Sciences, Faculty of Allied Health Sciences, University of Sri Jayewardenepura, Nugegoda, Sri Lanka

**Keywords:** Medication errors, Dispensing errors, Pharmacists, Sri Lanka

## Abstract

**Background:**

Dispensing errors, known to result in significant patient harm, are preventable if their nature is known and recognized. However, there is a scarcity of such data on dispensing errors particularly in resource poor settings, where healthcare is provided free-of-charge. Therefore, the purpose of this study was to determine the types, and prevalence of dispensing errors in a selected group of hospitals in Sri Lanka.

**Methods:**

A prospective, cross sectional, multi-center study on dispensing errors was conducted, in a single tertiary care, and two secondary care hospitals, in a cohort of 420 patients attending medical, surgical, diabetic and pediatric clinics. The patients were selected according to the population size, through consecutive sampling. The prescription audit was conducted in terms of dispensing errors which were categorized as i) content, ii) labelling, iii) documentation, iv) concomitant, and v) other errors based on in-house developed definitions.

**Results:**

A total of 420 prescriptions (1849 medicines) were analyzed (Hospital-I, 248 prescriptions-1010 medicines; Hospital-II, 84 prescriptions-400 medicines; Hospital-III, 88 prescriptions-439 medicines), and a cumulative total of 16,689 dispensing errors (at least one dispensing error in a prescription) were detected. Labelling errors were the most frequent dispensing error (63.1%; *N* = 10,523; *Mostly missing information on the dispensing label*), followed by concomitant prescribing and dispensing errors (20.5%; *N* = 3425; *Missing prescribing information overlooked by the pharmacist*), documentation errors (10.6%; *N* = 1772 *Missing identification of pharmacist on dispensing label),* clinically significant medication interactions overlooked by pharmacists (0.5%; *N* = 82), content errors (4.9%; *N* = 812; *Discrepancies between medication dispensed and prescription order),* medications dispensed in unsuitable packaging (0.4%; *N* = 74), and lastly medication dispensed to the wrong patient (0.01%; *N* = 1).

**Conclusions:**

Dispensing errors are frequent in Sri Lankan hospitals which operate with limited resources and provide free healthcare to all citizenry. Over one half of the errors were labeling errors with minimal content errors. Awareness on common types of dispensing errors and emphasis on detecting them could improve medication safety in Sri Lankan hospitals.

## Background

Patient management is a complex set of activities, and involves several healthcare professionals with diverse knowledge and skills. Once a disease diagnosis is made therapeutic intervention is the frequent norm, which entails appropriate drug prescribing, dispensing and administration of medications to achieve the desired outcomes. During this medication process, unintentional errors which are harmful or life-threatening to patients may occur, and these are termed medication errors. Medication errors are defined as failures in the treatment process that leads to or has the potential to cause harm to patients [1].

Dispensing itself is a complicated process [2] and involves receiving the prescription, interpreting and reviewing the appropriateness of prescribed medications, calculating required doses, preparing dispensing labels, retrieving and preparing medications, double-checking against the prescription, and handing over to patient with required instructions and counseling. The dispensing process could be carried out by a single pharmacist or by a team depending on the available resources. Dispensing errors could happen in both these instances resulting in numerous consequences [3–5]. There are accepted standards which should be maintained in each dispensing step to prevent errors [6]. These standards vary depending on the jurisdiction, where the process may either be automated, semi-automated or manually performed. In most jurisdictions, it is a legal requirement for dispensing to be performed under the supervision of a registered pharmacist either with or without the involvement of dispensers or automation [7–9].

Dispensing errors have been identified and defined in the literature with varying terminology and interpretations [2, 10, 11]. Some define a dispensing error simply as a discrepancy between medicines prescribed and medicines received by the patient [10] or as a discrepancy between the written order and the completed prescription [11]. Others have dichotomously sub-classified dispensing errors as either preventable or unpreventable errors [12–15], Thus, the errors detected after the medication had been issued and patient had left the pharmacy are classified as external errors/ incidents classified as unpreventable dispensing incidents/errors [12] [13] and second, as errors detected within the pharmacy before the issue of medications to patients that are termed near-misses, internal errors [12], or preventable dispensing incidents/errors [13]. However, numerous workers have used a more detailed classification of based on the type of the error (e.g. Wrong drug, wrong dose, wrong strength) [4, 13, 15–20] and then sub-grouped taking into consideration the specific temporal step at which the error was introduced into the process, as content errors [19, 20], labeling errors [19, 20], documentation errors [18], issue errors [4] and filing errors [17].

Although dispensing errors are unintentional, it is important to identify their prevalence and types, in order to minimize or to prevent them [16]. Several studies have been conducted in the West to investigate factors that cause dispensing errors [2, 16, 21] and outcomes of these studies have been subsequently used to improve medication safety in these settings. High workload, inadequate and inexperienced staff, illegible handwriting, physical distractions and interruptions in the working environment, and similar medication names (Look-alike/sound-alike medicine names) were found to be the common causes of dispensing errors [11, 22–24]. However, it must be noted that the nature and causes of dispensing errors may differ depending on the type of the healthcare setting and should not be generalized.

Dispensing errors impart an additional burden on healthcare systems [4]. The occurrence of ADEs/ADRs and drug interactions due to dispensing errors have been associated with significant clinical impact [4, 25]. Even though there is obvious financial burden in managing consequences of dispensing errors, there are no studies, to our knowledge, that clearly assess this aspect separately for dispensing errors, but as a cumulative consequence of medication errors [19, 20, 26–28]. Middle-income developing countries such as Sri Lanka lack adequate resources, both financial and human, to cope with this additional demand due to dispensing errors and consequent adverse drug events.

In Sri Lanka, healthcare services offered by the State is free-of-charge to its citizenry. More than 80% of the population benefit from this free service where, 54 million patients attend the outpatient departments annually, and 25.8 million patients attend various clinics of State hospitals, with integrated pharmacy services [29]. Owing to the stringent economic conditions and the non-profit services provided, the Sri Lankan healthcare system operates with major resource limitations, with prescriptions limited to a government mandated Essential Medicines List [24, 30, 31]. Given these concerns, an error-free, and a safe dispensing environ in outpatient care is an absolute necessity in hospitals, especially because dispensing is the last stage of the medication delivery process, and uncertifiable errors committed at this stage have a high likelihood of impacting patient well-being.

Although there are several Sri Lankan studies in reporting on prescription errors and medication errors [30, 32–35] only one investigation, to our knowledge, has reported some aspects of dispensing errors, their types and prevalence. The latter by Hettihewa et al. [36] reported a low dispenser to patient ratio, and the need for introducing a well-prepared medicine labeling system but they did not report detailed features of dispensing errors. Hence, in the absence of a basic data base on this subject, the current study was focused on acquiring much needed information on types and prevalence of dispensing errors in resource limited, free of charge healthcare systems like Sri Lanka.

## Methods

### Study design and setting

This study was a prospective cross sectional and multi-center study. Three State hospitals representing three different Provinces in Sri Lanka were selected for study through convenience sampling. Study hospital 1 (SH1) is the largest tertiary care hospital in the Uva Province with 1493 beds, and 40 medical consultants, serving 295 daily inpatient admissions, 810 daily outpatients and 424,024 annual clinic visits. Study hospital 2 (SH2) is a Type B Base Hospital (A hospital with all basic specialties, medical, diabetic, psychiatric, pediatric and several other clinics) in the Sabaragamuwa Province with 325 beds, and 12 medical consultants serving 120 daily inpatient admissions, 700 daily outpatients, and 126,272 clinic visits per year. Study hospital 3 (SH3) is a Type A Base Hospital (A hospital with all basic specialties, medical, diabetic, psychiatric, pediatric and several other additional specialties like dermatology, ophthalmology) in the North Western Province with 374 beds and 19 medical consultants, serving 175 daily admissions, 740 daily outpatients, and 132,000 annual clinic visits. There were 14 pharmacists involved in dispensing in SH1, four in SH2, and five in SH3. The cumulative population size of all three hospitals was approximately 52,942 patients (SH1, 31,336; SH2, 10,606; SH3, 11,000) per month.

### Study participants

The study cohort were both men and women, attending medical, diabetic, psychiatric or pediatric clinics in select three study hospitals, prescribed with at least one medicine from the relevant clinic, and obtaining medicines from the hospital pharmacy at the study site. Caregivers or family members of patients who had come to collect medicines from the pharmacy and had the prescription in hand were also eligible. Those prescribed with external preparations and medical devices only, and patients attending only for counselling sessions at psychiatric clinics were not considered for selection.

### Sample size calculation and sample selection

The required sample size was calculated using the Raosoft online sample size calculator (Raosoft. Inc), considering a 5% margin of error, 95% confidence level, 50% response distribution and a population size of 52,942. The calculated sample size was 380, but 420 was considered after accounting for 10% missing data. The calculated sample size was proportioned to the three hospitals based on the outpatient participation of each hospital in each month (SH1, *N* = 248; SH2, *N* = 84; SH3, *N* = 88). The sample size allocated for each hospital was further proportioned to each clinic based on the number of registered clinic attendees during each month.

### Data collection

A consecutive sampling method was used to select patients from each hospital. Data were collected every clinic day, on a selected month, in the morning from 10.00 a.m. to 12.00 noon from morning clinics, and from 2.00 p.m. to 4.00 p.m. from evening clinics until the sample size was achieved. At 10.00 a.m. the first patient available at the first dispensing counter was selected, and the next available patient in the adjacent counter at the time of completing the review of the first patient, was selected next.

### Study procedure

The prescription of a patient was matched with the corresponding dispensing labels and dispensed medicines to detect dispensing errors. Medication names, doses, frequencies, duration, number of units to be dispensed, and routes of administration, mentioned on the prescription, were matched with instructions written on the dispensing label and the medications dispensed to patient, to ensure accuracy. Further, a list of in-house definitions for dispensing errors was developed (See Supplementary Table 1, Additional file 1) and was used as a guide for identification of other dispensing errors. In-house definitions for dispensing errors were developed according to published literature [11, 16, 37, 38] and to suit the study setting (See Supplementary Table 1, Additional file 1). Three different pharmacists collected data in the three study settings but all were pre-trained on identification of dispensing errors.

Prior to collecting data, and for internal standardization, the three pharmacists who collected the data assessed dispensing errors in 10 hypothetical cases and compared results for discrepancies. All discrepancies were discussed until a final consensus was reached on how to proceed when similar situations are encountered. The British National Formulary [39], the Australian Medicines Handbook [40], and the Australian Pharmaceutical Formulary and Handbook [41] were used as references during data collection. Drug interactions were detected using an online drug interaction checker (Drugs.com) [42]. Drug interactions that were undetected by the dispensing pharmacist at the point of dispensing were considered as errors.

Where dispensed medicines or instructions on dispensing labels did not match with the respective prescription, the research pharmacist informed the dispensing pharmacist of this discrepancy and documented reasons where relevant. However, the research pharmacists did not provide any further interventions to correct dispensing errors which were detected.

### Statistical analysis

Data were analyzed using the statistical data analysis package, SPSS (Version 25.0). Results were presented as frequencies and percentages. Dispensing errors of the three hospitals were not compared as it was not an objective of our study. Chi square was used to compare proportions of dispensing errors among clinic types.

### Ethical consideration

Ethical approval for this study was obtained from the Ethics Review Committee of the University of Sri Jayewardenepura (Ref:85/17). Permission was obtained formally from all study hospitals. Written informed consent was obtained from study participants. Confidentiality of the data and the privacy of the participants were maintained through anonymity.

## Results

A total of 420 prescriptions, and 1849 medicines were evaluated from all the three hospitals (SH1, *N* = 248; SH2, *N* = 88; SH3, *N* = 84). There was a mean of 4.4 medications (SD 2.3) per each prescription (Ranging from 1 to 12 medications per prescription) (Table 1).

**Table 1:** Summary of prescriptions and medications analyzed

	SH1	SH2	SH3	Total
Number of prescriptions	248	84	88	420
Number of medicines	1010	400	439	1849
Mean number of medicines in a prescription (SD)	4.1 (2.3)	4.7 (2.5)	5.0 (2.3)	4.4 (2.3)
Total number of dispensing errors detected	9326	3036	4327	16,689

(Total number of prescriptions analyzed was used as the denominator to analyze the average number of medications in a prescription. *SD* Standard Deviation)

After evaluation of the prescriptions, a cumulative total of 16,689 dispensing errors were identified (Table 2). This high number of dispensing errors included labelling errors (Mostly missing information on the dispensing label, 63.1%; *N* = 10,523; approx. six labelling errors for each medication dispensed), concomitant prescribing and dispensing errors (Missing prescribing information overlooked by the pharmacist, 20.5%;*N* = 3425, and clinically significant medication interactions in prescriptions overlooked by pharmacists, 0.5%;*N* = 82; approx. two concomitant errors for each medication dispensed), documentation errors (Missing identification of pharmacist on dispensing label, 10.6%;*N* = 1772; approx. one documentation error for each medication dispensed), content errors (Discrepancies between medication dispensed and prescription order, 4.9%;*N* = 812; approx. 0.4 content errors for each medication dispensed), medications dispensed in unsuitable packaging (0.4%;*N* = 74; 0.04 packaging errors for each medication dispensed), and one case of medication dispensed to the wrong patient (0.01%) (Table 3). Dispensing error rates of each subtype are indicated in Table 3. See & insert Results (Table 3).

**Table 2:** Summary of different types of dispensing errors in study hospitals

	SH 1 (*N* = 248 prescriptions)	SH 2 (*N* = 84 prescriptions)	SH 3 (*N* = 88 prescriptions)	Total (*N* = 420 prescriptions)
Labelling errors, N (%)	6146 (65.0%)	1644 (56.5%)	2733 (63.2%)	10,523 (63.1%)
Concomitant prescribing and dispensing errors, N (%)	1878 (19.9%)	771 (24.4%)	858 (19.8%)	3507 (21.0%)
Documentation errors, N (%)	948 (10.0%)	400 (13.7%)	424 (9.8%)	1772 (10.6%)
Content errors, N (%)	477 (5.0%)	87 (3.0%)	248 (5.7%)	812 (4.9%)
Other errors, N (%)	6 (0.1%)	9 (0.3%)	60 (1.4%)	75 (0.4%)
Total	9455 (56.7%)	2911 (17.4%)	4323 (25.9%)	16,689 (100.0%)

(Total number of errors encountered from each hospital was used as the denominator to calculate column percentages. N, Number of prescriptions analyzed. Other errors = Errors made in the dispensing process which are not content, labelling, documentation or concomitant prescribing and dispensing errors)

**Table 3:** Details of nature and prevalence of dispensing errors in study hospitals

	Example	SH1 (*N*=1010)	SH2 (*N*=400)	SH3 (*N*=410)	Total (*N*=1849)	Error for each medication dispensed
Labelling errors						
Duration of medications not indicated on dispensing label	Diclofenac sodium tablets 50 mg bd for 3 days was prescribed. Six tablets were dispensed with directions to be used (one tablet two times per day) but without indicating that the treatment should continue for 3 days.	966	379	413	1758	1758/1849 = 0.95
Total quantity of medication dispensed not indicated on dispensing label	84 tablets of metformin (500 mg tds for 4/52) was dispensed without indicating the total number of tablets (84) on the dispensing label. 56 beclomethesome capsules (400 microgram BD 01 month) was dispensed without indicating the total number of capsules as 56 on the dispensing label.	958	216	416	1590	1590/1849 = 0.86
Dosage form is not indicated on dispensing label	Dispensed amoxicillin 125 mg chewable tablets and indicated amoxicillin 125 mg instead of amoxicillin 125 mg chewable tablets on the dispensing label	777	221	286	1284	1284/1849 = 0.69
Incorrect or incomplete medicine strength on dispensing label	Indicated thyroxin 50 mg instead of 50 micrograms on dispensing label	906	96	278	1280	1280/1849 = 0.69
Medicine strength not indicated on dispensing label	Prescribed aspirin 75 mg nocte and dispensed 28 tablets of aspirin 75 mg tablets in dispensing label indicating only ‘aspirin 01 at night’ instead of ‘aspirin 75 mg take 01 tablet at night’	907	61	284	1252	1252/1849 = 0.68
Incorrect or incomplete medicine name (using unapproved abbreviations) on dispensing label	Indicating paracetamol as PCM, carbamazepine as CBZ on dispensing label	508	221	288	1017	1017/1849 = 0.55
Medicine name not indicated on dispensing label (neither generic nor brand)	Verapamil 40 mg tds was prescribed and 84 tablets of verapamil was dispensed with directions to be used, but without indicating the medication name on the dispensing label Was commonly observed with paracetamol and chlorpheniramine as well	514	34	198	746	746/1849 = 0.40
Incorrect or incomplete dosage form on dispensing label	Indicating ISMN 60 mg only instead of ISMN 60 mg SR tablet on dispensing label	233	179	153	565	565/1849 = 0.31
Special instructions not provided where necessary	Instruction of ‘Take at least half an hour before food’ was not on the dispensing label for omeprazole. Swallow whole (Do not crush or chew) for enteric coated tablets such as erythromycin and omeprazole was absent.	241	117	151	509	509/1849 = 0.28
Failing to attach auxiliary labels	Additional labels of “Shake the bottle” and “Store in refrigerator” was not attached to reconstituted cephalexin syrup container (Cephalexin was reconstituted in bulk and the required volume was dispensed in a different container without original label indicating these information)	127	83	73	283	283/1849 = 0.15
No label with dispensed medicine	Paracetamol 2 tbs SOS was prescribed and 20 paracetamol tablets were dispensed in an envelope with no written information on the envelope. Same was observed with salbutamol and beclomethasone capsules. Insulin 12 IU mane and 10 IU nocte was prescribed and 1 vial of insulin has dispensed in a container without a dispensing label.	-	21	153	174	174/1849 = 0.09
Dosing intervals and frequency not indicated on dispensing label	Paracetamol two tablets’ written instead of ‘paracetamol two tablets to be taken every 6 hrly Dry powder capsules of salbutamol and beclamethasone as prescribed as 1 capsule bd and it was dispensed to patients without any dosing interval or frequency of administration Was not with dry powder capsules of salbutamol (Asthelin)	09	16	40	65	65/1849 = 0.04
Total		**6146**	**1644**	**2733**	**10523 (63.1%)**	
Concomitant errors						
Medicine name, route, dosage form not indicated in prescription but ignored by pharmacist	Losartan 1 bd was written instead of losartan 50 mg tablet bd for 1/12	922	381	418	1721	1721/1849 = 0.93
Prescriber not identified in prescription but ignored by pharmacist	-	918	316	422	1656	1656/1849 = 0.90
Clinically significant drug interactions on prescription missed by pharmacist	Medicines have been dispensed without detecting the drug-drug interactions (Table 4) in the prescription Eg: Both enalapril and spironolactone were prescribed together and the interaction was not detected by the pharmacist. Both medicines were dispensed to be used together	38	26	18	82	82/1849 = 0.04
Patient name and age not indicated in prescription but ignored by pharmacist	-	-	48	-	48	48/1849 = 0.03
Total		**1878**	**771**	**858**	**3507 (21.0%)**	
Documentation errors						
Pharmacist who dispensed the medications were not indicated on label	-	948	400	424	1772	1772/1849 = 0.96
Total		**948**	**400**	**424**	**1772 (10.6%)**	
Content errors						
Wrong number of units	Issuing 31 tablets of atorvastatin 10 mg instead of 28 tablets (03 tables were issued in excess)	462	78	139	679	679/1849 = 0.37
Wrong dosage form	Dispensing a slow release form of ISMN 60 mg SR instead of normal release ISMN 30 mg. (Patient was advised to crush it and take the half from ISMN 60 mg SR)	15	07	44	66	66/1849 = 0.04
Wrong strength	Dispensing of hydrochlorothiazide 25 mg tablets instead of 50 mg when prescribed as 1 tablet in the prescription	-	02	60	62	62/1849 = 0.03
Wrong medications	Dispensing of famotidine instead of omeprazole at verbal request of the prescriber but not corrected in the prescription	-	-	05	05	05/1849 = 0.003
Medication omissions	[No errors detected]	-	-	-	-	
Deteriorated medicine	[No errors detected]	-	-	-	-	
Total		**477**	**87**	**248**	**812 (4.9%)**	
Other errors						
Medications dispensed in unsuitable packaging	Glyceryl trinitrate (GTN) and thyroxin were dispensed in a container without light protection	06	08	60	74	74/1849 = 0.04
Medications dispensed to wrong patient	Patient was found carrying medications which were left behind in the counter by the previous patient	-	01	-	1	01/1849 = 0.0005
Total		**06**	**09**	**60**	**75 (0.4%)**	
		**9455**	**2911**	**4323**	**16689**	

Total number of dispensing errors in each category was used as denominator to calculate column percentage

*N* Number of dispensing errors

There were five incidents of dispensing wrong medicines where famotidine was dispensed when omeprazole was written on the prescription but on verbal instructions by the prescriber. The prescription was not corrected accordingly and there was no systematic procedure to record verbal instructions received from the prescriber. The wrong strength of medicine was dispensed to 62 patients including dispensing of hydrochlorothiazide 25 mg when ‘1 tab’ (one tablet) was written on the prescription. Further examination of patient records revealed that the intention was to prescribe 50 mg and not 25 mg. The latter incident was a good example of a concomitant prescribing and dispensing error due to the prescriber not indicating the strength of the medicine. Further, the pharmacist had not attempted to clarify the prescription prior to dispensing the medication, and had dispensed the strength of the medicine available in the pharmacy.

Wrong dosage form errors were mainly due to dispensing of modified dosage forms instead of normal release forms which were expected to be administered by breaking or crushing (*N* = 62). Aspirin 150 mg enteric coated tablets were dispensed instead of aspirin 75 mg tablets (to be broken in half), and sodium valproate 200 mg enteric coated tablets were dispensed instead of 100 mg tablets (to be broken in half). Dispensing the wrong number of medication units was the most prominent content error recorded in all three hospitals. One or two tablets/capsules were issued in excess of the prescribed amount in many instances (Table 3) See & insert Results (Table 3).

Prevalence of labeling errors were significantly higher (*P* < 0.001) in pediatric clinics compared to other types of clinics (See Supplementary Table 2, Additional file 2). It was observed that labelling errors such as missing name of the medicine and missing dosage form, were reduced significantly when using printed, as opposed to handwritten, labels (*P* < 0.001). However, labeling errors such as failing to include the duration of medications in the label persisted even with printed labels (No significant difference between printed and handwritten label, *P* = 0.171).

Eighteen clinically significant drug interactions were detected among 82 prescribed medications (Table 4) and most were related to the psychiatry clinics (40/82). Only one incident of dispensing to a wrong patient was detected where a patient had picked a medication pack accidently left behind by another patient.

**Table 4:** Clinically significant drug – drug interactions in prescriptions which were not detected by pharmacists

Interacting medicine pair	Severity of interaction^a^	Frequency (%)
Olanzapine, clonazepam	Major	11 (27.5)
Clopidogrel, omeprazole	Major	3 (7.5)
Amitriptyline, fluoxetine	Major	3 (7.5)
Haloperidol, fluphenazine	Major	3 (7.5)
Olanzapine, topiramate	Major	3 (7.5)
Losartan, spironolactone	Major	2 (5.0)
Haloperidol, promethazine	Major	2 (5.0)
Haloperidol, lithium	Major	2 (5.0)
Haloperidol, chlorpromazine	Major	2 (5.0)
Enalapril, spironolactone	Major	1 (2.5)
Fluoxetine, clopidogrel	Major	1 (2.5)
Imipramine, haloperidol	Major	1 (2.5)
Pioglitazone, clopidogrel	Major	1 (2.5)
Sodium valproate, lamotrigine	Major	1 (2.5)
Captopril, potassium chloride	Major	1 (2.5)
Imipramine, fluoxetine	Major	1 (2.5)
Enalapril, potassium chloride	Major	1 (2.5)
Clonazepam, topiramate	Major	1 (2.5)
**Total**		**40**

^a^Severity of interactions as denoted in the Drugs.com [25], online drug interaction checker

## Discussion

In total we assessed 420 prescriptions that encompassed 1849 medications, from three Sri Lankan hospitals, and noted 16,688 dispensing errors. Amongst the 1849 medications, labelling errors (*N* = 10,523), concomitant prescribing and dispensing errors (*N* = 3507), documentation errors (*N* = 1772), content errors (*N* = 812), medications dispensed in unsuitable packaging (*N* = 74), and one case of medication dispensed to the wrong patient were identified. It was evident that, most dispensing errors were only potential errors related to missing information in dispensing labels.

It was interesting, and somewhat disappointing to note that all of the 420 prescriptions we evaluated had at least one dispensing error. In a similar study from Brazil, Anacleto et al., (2007) also reported at least one dispensing error in 81.8% of prescriptions, after evaluating 345 prescriptions [22]. However, most studies have reported a lower prevalence of errors relative to the current study. Thus, an American study in 2003 reported a dispensing error rate of 3.6% (among 5075 prescriptions, 140,755 medication doses) [4], a study in France in 2009, a rate of 2.5% (among 734 unit dose medication cassettes) [42], and a study in UK in 2002, a rate of 2.1% (among 849 dispensed items) [24]. However, it was difficult to assess if all these studies had used the same explicit definitions as we did to justify comparison. A review of dispensing errors also revealed that dispensing error rates varied between countries (0.015–33.5%) depending on the dispensing system, research method, and classification of dispensing error types [43]. Our study examined the full range of errors that could occur during the dispensing process including missing prescriber and pharmacist identification, on prescriptions and dispensing labels, respectively.

Labeling errors, documentation errors, content errors and concomitant (prescribing and dispensing) errors were the dispensing error types assessed by most other studies [11, 22, 43, 44]. While content errors were most frequent in other reported studies [4, 12, 42] labelling errors (63.1% *n* = 10,523) were the highest in the current study. Nevertheless, similar findings have also been reported in developed countries like USA where Flynn and Breger (1999) reported 80% of dispensing errors to be wrong labelling information in the ambulatory care setting [45], and James et al., (2008) reported a labeling error rate of 58.2% followed by 41.8% of content errors in a Welsh (UK) national hospital setting [2].

All content errors could cause serious harm to patients. In our study, among the 4.9% content errors, most were related to dispensing the wrong number of medication units (83.8%; *n* = 540)) where one or two tablets were issued in excess of the prescribed quantity. Although this seems a trivial mistake, consequences of dispensing extra doses to patients could be harmful. Besides, wastage of medicines will undoubtedly add to the healthcare cost especially in limited resource settings like Sri Lanka. A similar pattern was also reported by Cina et al., (2006) in USA where the highest dispensing errors reported were related to supply of the wrong quantity of medications (59%, *n* = 2970) [4]. Even among the few content errors found in this study, wrong medication and wrong strength errors were minimal (9 and 11% respectively) [4]. In contrast, most common content errors reported in UK were wrong medications (23.0%), wrong strength (23.0%), wrong directions (10.0%) or wrong quantity (10.0%) errors in 2002 [46]. Another UK study by Beso et al., in 2005 found missing doses to be the most frequent (*n* = 16), followed by incorrect strength (*n* = 15) and incorrect medication (*n* = 11) errors, among a total of 70 (54%) content errors and 130 dispensing errors [16]. In Thailand, the dispensing error rate was 1.68 per 10,000 prescriptions in State hospitals and 55.7% of them were wrong medications, 19.4% were wrong strength and 10.0% were wrong dosage form errors [44].

It is also noteworthy that the labeling errors we found were mostly missing information compared to incorrect information, as reported by others [2–4, 16]. Of the missing information, duration of treatment (*N* = 1758, 16.7%), quantity of medication dispensed (*N* = 1590, 15.1%), dosage form (*N* = 1284, 12.2%) and medication strength (*N* = 1280, 12.1%) were the most frequent. In contrast, others have mostly reported wrong medication details on the label [47, 48].

Hettihewa et al., (2011) reported that pharmacists in Sri Lanka spent less than a minute (0.81 min) on dispensing medications to a patient and this could be the major reason for many labelling errors [36]. A single pharmacist must cater for many patients which limits the time spent on one patient [49] and can be a reason for omitting details on handwritten dispensing labels. Although missing information in labels may not cause immediate harm, such malpractices could lead to issues such as patient non-compliance, overdose due to duplications, and sub-optimal outcomes. Hence system errors of this nature must be given due importance to minimize dispensing errors.

Prescribing errors not detected by pharmacists were categorized as concomitant errors (Concomitant prescribing and dispensing errors). We reported 18 clinically significant drug interactions which were missed by pharmacists, which must be given serious consideration. In addition, this category also contained errors with no immediate harm such as incompleteness of prescriptions which the pharmacist ignored (*N* = 3427/3507). Silva et al., (2008) found 615 (87.9%) medicines dispensed without the dosage form being specified in a Brazilian hospital. While the prescriber could not be identified in 8.4% of prescriptions dispensed in Brazil [50], we reported similar results where 10.4% of prescriptions had missing prescriber identification. Potential errors such as missing information could lead to fatal consequences and hence should be corrected through system improvement.

There are a few noteworthy limitations in the current study. For instance, though a multi-center investigation, only three hospitals were selected through convenience sampling, thus limiting the generalizability of our results to the whole country. Three research pharmacists independently collected data in the three study settings, and despite their training, there may have been slight variations in identifying dispensing errors. It was also possible that most of the missing instructions in dispensing labels were given verbally by pharmacists (only the written instructions were considered), as this element could not be assessed due to the retrospective nature of the study. Harm caused by dispensing errors were not monitored, hence potential harms such as incapacities and hospitalization could not be evaluated in this study. Though this was a multi-centered study results of each study setting were not compared as our objective was only to assess the general prevalence and types of dispensing errors encountered in hospitals in Sri Lanka.

## Conclusions

This study was on dispensing errors conducted in three State hospitals in Sri Lanka using explicit definitions and methodology to grasp all aspects of dispensing errors. Among 1849 medications, 16,689 dispensing errors were detected which included, labelling errors (63.1%; *N* = 10,523), concomitant prescribing and dispensing errors (21.0%;*N* = 3507), documentation errors (10.6%;*N* = 1772), content errors (4.9%;*N* = 812), medications dispensed in unsuitable packaging (0.4%;*N* = 74), and one case of medication dispensed to the wrong patient. Of the many dispensing errors observed, most were related to missing information in dispensing labels which reflects a poor system, leading to potential harm. Discrepancies between medication dispensed and prescription order (content errors) were minimal in contrast to other published research on dispensing errors. Concomitant prescribing and dispensing errors also existed indicating that pharmacists failed to detect some prescribing errors.

It is highly recommended that all pharmacists are made aware of dispensing errors that happen including the system related errors that could lead to patient harm. Dispensing errors that could lead to medication waste should also be tackled in resource limited healthcare systems such as Sri Lanka. Streamlining and formalizing the dispensing process is essential for developing countries as this could help avoid many dispensing hazards that could arise due to resource limitations. The standard of prescriptions should be improved through continuous awareness programs. We recommend national guidelines on dispensing of medicines to be developed to suit resource limited settings and to be implemented across the country.

## Supplementary Information

**Additional file 1: Supplementary Table 1**: Important supplementary table with results was with document named as Supplementary Table 1: In-house definition of dispensing errors.

**Additional file 2: Supplementary Table 2**: Important supplementary table with results was with document named as Supplementary Table 2: Prevalence of labeling errors clinic and hospital wise.

## Data Availability

The datasets used and/or analyzed during the current study are available from the corresponding author on reasonable request.

## Abbreviations

UK: United Kingdom
USA: United State of America
ADE: Adverse drug event
ADR: Adverse drug reaction
