# Supplementary material for: Nature of dispensing errors in selected hospitals providing free healthcare: a multi-center study in Sri Lanka
Source: BMC Health Serv Res. 2020 Dec 14;20:1140. doi: 10.1186/s12913-020-05968-y (PMC7734753; doi:10.1186/s12913-020-05968-y)
Supplement: Supplementary file 1 — Additional file 1: Supplementary Table 1: Important supplementary table with results was with document named as Supplementary Table 1: In-house definition of dispensing errors. [file 12913_2020_5968_MOESM1_ESM.docx]

**Additional file 1**

**Supplementary Table 1**

**Supplementary Table 1: In-house definition of dispensing errors**

| **Type of Dispensing error** | **Definition** | **Subcategory** | **Definition** |
| --- | --- | --- | --- |
| **Content errors** | Discrepancies between the content of medicine dispensed and the prescription order | **Wrong drug** | Dispensed medicine is different from the medicine prescribed in the order/prescription, or a medicine which is not in the prescription has been dispensed |
|  |  | **Omissions** | A medicine has not been dispensed (missed) even though it has been prescribed |
|  |  | **Wrong dose** | The medicine has been dispensed at a higher or lower dose than that specified in the order/prescription, or the medicine was prescribed and dispensed without the strength being specified in the prescription |
|  |  | **Wrong dosage form** | The dosage form has been dispensed in a form which is different to that prescribed |
|  |  | **Wrong number of units** | The total number of units dispensed is different to that specified in the prescription |
|  |  | **Expired/deteriorated drug** | The medicine dispensed has altered physical, organoleptic characteristics that were recognizable to the necked eye during dispensing, expiration date absent, or observe damage to the original packaging (E.g. blister pack) |
| **Labeling errors** | Discrepancy between the label of medicine dispensed and the prescription order  Or  Missing essential labeling information | **Wrong/ No medicine name on label** | The medicine has been dispensed with a wrong name, or unacceptable abbreviation on the label or without a name of the medicine on the label |
|  |  | **Wrong/ No strength on label** | The medicine has been dispensed with a wrong strength on the label or without a medicine strength on the label |
|  |  | **Wrong/No dosage form on label** | The medicine has been dispensed with a wrong dosage form on the label or without a dosage form on the label |
|  |  | **Wrong/No ancillary label** | The medicine has been dispensed with a wrong warning label, without a warning label, when such a label is required* |
|  |  | **Wrong/No quantity on label** | The medicine has been dispensed with a wrong quantity indicated on the label or without a quantity on the label |
|  |  | **Completely wrong label or No label on package** | The medicine has been dispensed with a completely wrong label or without a label on the package |
|  |  | **Wrong frequency on label** | The medicine has been dispensed without or with incorrect dose intervals to what has been prescribed |
|  |  | **Wrong/No duration on label** | The medicine has been dispensed with a wrong duration on the label or without a duration on the label |
|  |  | **Absence of instructions** | The medicine has been dispensed without additional information/instructions* |
| **Documentation errors** | The name of the pharmacist who dispensed medicines was poorly legible, illegible or absent in the copy of the prescription order form retained in the pharmacy and prescription order forms given to the patient (eg: medicine order forms in clinic books) | | |
| **Concomitant errors** | Prescribing errors missed by the pharmacists | **Clinically significant drug interactions on prescription not detected by pharmacist** | Medicines with clinically significant interactions identified by drugs.com (26) has been prescribed and dispensed without being detected |
|  |  | **Wrong doses on prescription, not detected by pharmacist** | The medicine has been prescribed in low or high doses than recommended and being dispensed without detecting |
|  |  | **Wrong duration on prescription, not detected by pharmacist** | The duration of the medicine is unacceptable but has been dispensed without correcting |
|  |  | **Incomplete prescription dispensed by the pharmacist** | The medicine has been dispensed to a prescription which is incomplete or illegible and has not been detected by pharmacist. Patient Information, prescriber information, medicine information was assessed in terms of completeness of prescription |
| **Other errors** | Errors made in the process of dispensing which is not included in any of the above four categories | **Issued to wrong patient** | The medicine has been issued to a person other than the one intended in the prescription |
|  |  | **Incorrectly packed** | The medicine is supplied without appropriate package or a container |

* P*resence/absence of the ancillary labels and the presence/absence of special instructions in written were detected as per the Australian Pharmaceutical Formulary and Handbook (APF) and Australian Medicine Handbook (AMH).*
