# Supplementary material for: Nature of dispensing errors in selected hospitals providing free healthcare: a multi-center study in Sri Lanka
Source: BMC Health Serv Res. 2020 Dec 14;20:1140. doi: 10.1186/s12913-020-05968-y (PMC7734753; doi:10.1186/s12913-020-05968-y)
Supplement: Supplementary file 2 — Additional file 2: Supplementary Table 2: Important supplementary table with results was with document named as Supplementary Table 2: Prevalence of labeling errors clinic and hospital wise. [file 12913_2020_5968_MOESM2_ESM.docx]

**Additional file 3**

**Supplementary Table 2**

**Supplementary Table 2: Prevalence of labeling errors clinic and hospital wise**

|  | SH 1 (N=6146) | | | |  | |  | | SH 2 (N=1644) | | | | | SH 3 (N=2733) | | | |  | | Total(N=10523) | | | |  | |  |
| --- | --- | --- | --- | --- | --- | --- | --- | --- | --- | --- | --- | --- | --- | --- | --- | --- | --- | --- | --- | --- | --- | --- | --- | --- | --- | --- |
| Labelling errors (N,%) | Medical | Diabetic | Psychiatric | Pediatric | |  | | Medical | | Diabetic | Psychiatric | Pediatric |  | Medical | Diabetic | Psychiatric | Pediatric | |  | Medical | Diabetic | Psychiatric | Pediatric | | Total | |
| No label on medicines | - | - | - | - | |  | | 9 | | 11 | - | 1 |  | 53 | 63 | 24 | 13 | |  | 62 | 84 | 24 | 14 | | 174 | |
|  | - | - | - | - | |  | | 6.0 % | | 6.5% | - | 4.5% |  | 27% | 46% | 32% | 38% | |  | 8.5% | 12% | 8.2% | 8.8% | | 9.9% | |
| Medicine name not indicated on dispensing label | 185 | 117 | 118 | 94 | |  | | 13 | | 9 | 01 | 11 |  | 82 | 70 | 28 | 18 | |  | 280 | 196 | 147 | 123 | | 746 | |
|  | 48% | 32% | 79% | 91% | |  | | 9 % | | 5 % | 1 % | 50% |  | 42% | 52% | 38% | 53% | |  | 39% | 29% | 51% | 77% | | 40 % | |
| Incorrect or incomplete medicine name on dispensing label | 138 | 142 | 131 | 97 | |  | | 70 | | 80 | 56 | 15 |  | 119 | 85 | 50 | 34 | |  | 387 | 307 | 237 | 146 | | 1017 | |
|  | 36% | 38% | 88% | 94% | |  | | 50% | | 48% | 81% | 68% |  | 60% | 63% | 68% | 100% | |  | 53% | 46% | 81% | 92% | | 58% | |
| Strength of the medicine not indicated on dispensing label | 369 | 304 | 133 | 101 | |  | | 30 | | 13 | 03 | 15 |  | 117 | 85 | 48 | 34 | |  | 516 | 402 | 184 | 150 | | 1252 | |
|  | 95% | 82% | 89% | 98% | |  | | 21% | | 8 % | 4 % | 68% |  | 59% | 63% | 66% | 100% | |  | 71% | 60% | 63% | 94% | | 68% | |
| Incorrect or incomplete medicine strength on dispensing label | 369 | 304 | 134 | 99 | |  | | 41 | | 20 | 20 | 15 |  | 117 | 85 | 42 | 34 | |  | 527 | 409 | 181 | 148 | | 1280 | |
|  | 95% | 82% | 90% | 96% | |  | | 29% | | 12% | 29% | 68% |  | 59% | 63% | 58% | 100% | |  | 73% | 61% | 62% | 93% | | 68 % | |
| Dosage form not indicated on dispensing label | 290 | 253 | 135 | 99 | |  | | 70 | | 80 | 56 | 15 |  | 118 | 85 | 53 | 30 | |  | 478 | 418 | 244 | 144 | | 1284 | |
|  | 75% | 68% | 91% | 96% | |  | | 50% | | 48% | 81% | 68% |  | 60% | 63% | 73% | 88% | |  | 66% | 62% | 84% | 91% | | 69 % | |
| Incorrect or incomplete dosage form on dispensing label | 108 | 62 | 48 | 15 | |  | | 94 | | 48 | 30 | 07 |  | 76 | 48 | 20 | 9 | |  | 278 | 158 | 98 | 31 | | 565 | |
|  | 28% | 17% | 32% | 15% | |  | | 67% | | 29% | 43% | 32% |  | 39% | 36% | 27% | 26% | |  | 38% | 23% | 34% | 19% | | 31 % | |
| Failing to add auxiliary label | 40 | 35 | 07 | 45 | |  | | 22 | | 40 | 11 | 10 |  | 24 | 22 | 13 | 14 | |  | 86 | 97 | 31 | 69 | | 283 | |
|  | 10% | 9% | 5% | 44% | |  | | 16% | | 24% | 16% | 45% |  | 12% | 16% | 18% | 41% | |  | 12% | 14% | 11% | 43% | | 15 % | |
| Total number of units not indicated on dispensing label | 378 | 339 | 140 | 101 | |  | | 68 | | 79 | 54 | 15 |  | 186 | 125 | 71 | 34 | |  | 632 | 543 | 265 | 150 | | 1590 | |
|  | 97% | 92% | 94% | 98% | |  | | 48% | | 47% | 78% | 68% |  | 94% | 93% | 97% | 100% | |  | 87% | 81% | 91% | 94% | | 86 % | |
| Dosing intervals and frequency not indicated on dispensing label | 2 | - | 07 | - | |  | | 10 | | 06 | - | - |  | 17 | 15 | 08 | - | |  | 29 | 21 | 15 | - | | 65 | |
|  | 0.5% | - | 4.7% | - | |  | | 7.1% | | 3.6% | - | - |  | 8.6% | 11.1% | 11% | - | |  | 4 % | 3.1% | 5.2% | - | | 3.5% | |
| Duration of medicines not indicated on dispensing label | 379 | 346 | 140 | 101 | |  | | 124 | | 164 | 69 | 22 |  | 187 | 122 | 70 | 34 | |  | 690 | 632 | 279 | 157 | | 1758 | |
|  | 98% | 94% | 94% | 98% | |  | | 88% | | 98% | 100% | 100% |  | 95% | 90% | 96% | 100% | |  | 95% | 94% | 96% | 99% | | 95 % | |
| Special instructions not provided where necessary | 100 | 117 | 20 | 24 | |  | | 44 | | 56 | 07 | 10 |  | 76 | 50 | 11 | 14 | |  | 220 | 209 | 38 | 42 | | 509 | |
|  | 26% | 32% | 13% | 23% | |  | | 31% | | 33% | 10% | 45% |  | 39% | 37% | 15% | 41% | |  | 30% | 31% | 13% | 26% | | 28 % | |
| Labelling errors per drug | 6.0 | 5.4 | 6.8 | 7.5 | |  | | 4.2 | | 3.6 | 4.4 | 6.1 |  | 5.9 | 6.3 | 6.0 | 7.8 | |  | 5.7 | 5.1 | 5.9 | 7.3 | | 5.7 | |

(*N= number of Labelling errors detected; Denominator used to calculate column percentage was the total number of medicines analyzed in each clinic at each study hospital)*
